# The influence of environmental factors on communities of arbuscular mycorrhizal fungi associated with *Chenopodium ambrosioides* revealed by MiSeq sequencing investigation

**DOI:** 10.1038/srep45134

**Published:** 2017-03-22

**Authors:** Xihui Xu, Chen Chen, Zhou Zhang, Zehua Sun, Yahua Chen, Jiandong Jiang, Zhenguo Shen

**Affiliations:** 1 College of Life Sciences, Nanjing Agricultural University, Nanjing 210095, People’s Republic of China

## Abstract

Arbuscular mycorrhizal fungi (AMF) affect multiple ecosystem functions and processes, the assemblages of which vary across ecosystems. However, the influences of environmental factors on AMF communities which may shape these communities are still largely unknown. In this study, AMF communities from roots and rhizosphere soils of *Chenopodium ambrosioides* in different natural soils were investigated. The root habitat showed significantly smaller numbers of OTUs and lower community richness compared to the rhizosphere soil habitat. Most OTUs in the root habitat were shared by the soil habitat from the same sampling site, indicating that rhizosphere soils represent a pool of AMF species, a fraction of which is recruited by plants. Most of the AMF in root habitats were Glomeraceae, suggesting recruitment preferences of AMF by plants. The relative contributions of environmental factors to explain variations in AMF community composition and phylogenetic structure were assessed. The results revealed soil properties predominantly explained the variation, followed by geographic and climate parameters which explained a small fraction independently, while the host plant showed few explanations. Overall, our results indicated that soil and root habitats as well as soil characters, especially pH, nitrogen and micronutrients (Zn and Cu) affected AMF communities significantly.

The arbuscular mycorrhizal fungi (AMF, Glomeromycota) can form mutualistic symbioses with most terrestrial plants^1^. AM mutualism is an ancient plant–microbe interaction, which evolved very early in the evolution of terrestrial plants and is considered to have been crucial for the successful colonization of land by plants^2^,^3^,^4^. This mutualistic symbiosis enhances plant nutrient acquisition from soil and enhances tolerance to diverse biotic and abiotic stresses^5^,^6^. For example, mycorrhizal plants exhibit greater uptake of water and mineral nutrients than non-mycorrhizal plants^1^; AMF protect host plants against heavy metals (HMs) and enhance their HM tolerance^7^,^8^.

Environmental parameters and host plant species could affect AMF communities in terms of their composition and distribution^9^,^10^,^11^,^12^,^13^. Cheng *et al*. reported that a higher soil phosphorus (P) level is associated with lower mycorrhizal root colonization rates and lower AMF diversity^14^. Various AMF communities colonize the same host plant depending on the soil P concentration, according to Gosling *et al*.^15^. Thus mineral nutrients are important environmental factors. Furthermore, soil pH has been suggested to have a marked effect on AMF communities in agroecosystems and crops^10^. It has also been revealed that soil texture rather than P may influence AMF community structure^16^. Several studies have focused on the influence of HMs on AMF communities^17^,^18^. However, most of these studies were confined to local geographical scales and a limited set of soil characters. Thus, it would be helpful to consider a greater number of parameters of environment from a wider geographical area to evaluate the effects of environmental factors on AMF communities.

Many case studies have relied on microscopic identification of AMF spores from the field^19^,^20^,^21^. However, some AMF species sporulate infrequently or not at all in the field, and so might not be identified^22^,^23^. Moreover, spore density in soils does not necessarily reflect the AMF population colonizing the plant roots^22^,^24^. Recently, molecular analyses of DNA extracted from soils or roots have been carried out to evaluate AMF communities^25^,^26^. High-throughput sequencing technologies, such as 454 and Miseq amplicon pyrosequencing, facilitate efficient characterization of AMF communities by sequencing specific amplicons^27^,^28^. These technologies enable more detailed analyses of root or soil AMF communities.

Here, we investigated AMF communities in the rhizosphere soils and the roots of *Chenopodium ambrosioides*, a common plant species with a broad distribution in China, to identify the factors that affect AMF communities among different areas and habitats. To accomplish this, we collected root and rhizosphere soil samples from different geographical regions. Using high-throughput sequencing of a fragment of the small subunit (SSU) rRNA gene, we evaluated the composition of AMF communities in both root and rhizosphere soil habitats and investigated the relationships between the compositions of AMF communities and soil physical, chemical, and biological characteristics.

## Results

### Soil chemical characteristics

The majority of the physicochemical characteristics differed significantly among soil types (Table 1). All soil samples were neutral (pH 7.04–7.62), with the exception of GP (pH 5.62). The XC sample had the highest NO_3_^−^-N level. The TL and WH samples had considerably higher OM and total N levels but lower NH_4_^+^-N level than the other samples. Although lower total P, K, and Zn levels were detected in SS, TL, and WH compared with DN, GP, and XC, the available P, K and Zn levels were similar among all of them. Both the available and total Mn concentrations in SS, TL, and WH were significantly lower than those of DN, GP, and XC. The location, soil physical properties, plant parameters and climate characters of all sampling sites are shown in Supplementary Table S1.

### Overall sequencing information and taxonomic richness

A total of 1,465,650 paired-end sequences were obtained from the 36 libraries using the AMV4.5NF/AMDGR primer set. These sequences were overlapped to obtain high-quality tag sequences, and the dominant length distribution was 200−250 bp (>93%), with an average length of 218 bp. The details of the tag sequences obtained from each of the 36 samples are provided in Supplementary Table S2.

The taxonomic distributions of the obtained sequences in rhizosphere soil and root samples are shown in Supplementary Fig. S1. Fungal sequences other than Glomeromycota were detected. However, the majority of fungal sequences amplified with AMV4.5NF/AMDGR belonged to Glomeromycota (705,323 sequences, corresponding to 60.1% of the total). The second and third largest groups were Basidiomycota (15.2%) and Chytridiomycota (15.1%), respectively. The lowest numbers of Glomeromycota sequences were obtained for root and soil samples of XC (R-XC and S-XC), while the percentage of Glomeromycota sequences obtained from other samples was >40%. The Glomeromycota sequences were extracted for further analyses. Specifically, pyrosequencing of XC3 and WH3 failed because few Glomeromycota sequences were obtained, and so these two samples were excluded from further analyses.

### AMF community richness and diversity

All of the rarefaction curves tended to reach saturation (Supplementary Fig. S2), revealing that the data volume of sequenced reads was sufficient to detect the majority of sequence types. Marked variation in total OTU number among the samples was suggested by the rarefaction curve. The samples from rhizosphere soil habitats had a larger number of OTUs (20.5–55.5) compared to those from root habitats (4.0–19.7) (Table 2). Consistently, the richness indices, Chao1 and ACE, showed that the samples from root habitats had the lowest number of AMF OTUs, while the samples from rhizosphere soil habitats had the highest number, with significant differences between habitats. However, no significant differences of Shannon diversity were detected between the root and rhizosphere soil habitats.

### AMF community composition

**Figure 1:**
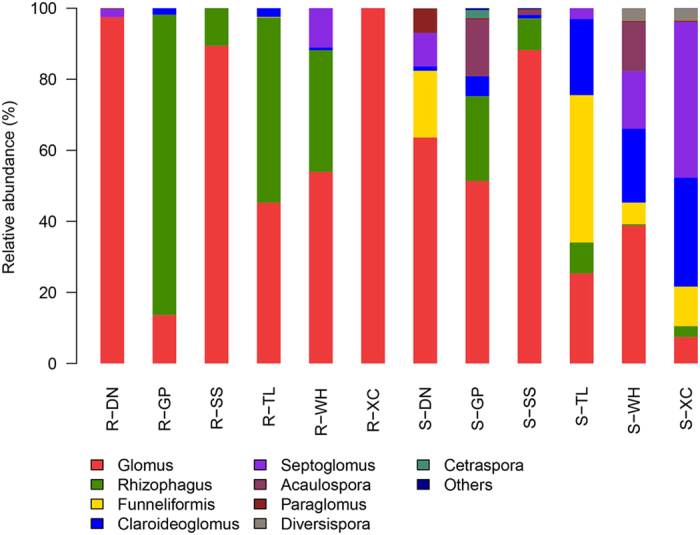
Abundance percentages of AMF genera for all samples. R, root samples; S, soil samples.

Significant variation in AMF community composition at the genus level was detected among both root and soil samples (Fig. 1). Sequences that could be classified were affiliated with nine AMF genera, while those that could not be classified were assigned as others. *Glomus* and *Rhizophagus* were the most abundant genera in all root samples, but their relative levels differed. A greater number of genera were present in samples from rhizosphere soil habitats; the most abundant genera were *Glomus, Funneliformis*, and *Claroideoglomus*.

**Figure 2:**
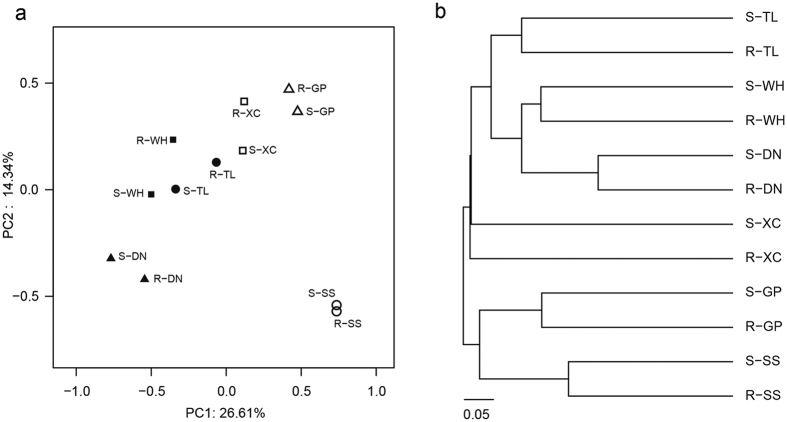
Principal coordinates analysis (**a**) and hierarchical clustering (**b**) with Bray–Curtis distances of AMF communities. R, root samples; S, soil samples.

A PCoA based on the OTU composition is shown in Fig. 2a. Of the variation in the AMF communities, 26.6% and 13.3% could be explained by the first and second principal components, respectively. The samples from root and soil habitats at the same site clustered together (Fig. 2a). This was confirmed by the hierarchical cluster analysis, which showed clusters of samples from the same site (Fig. 2b).

Indicator species analyses were used to identify specific OTUs associated with the rhizosphere soil or root habitat. Sixteen OTUs were found in rhizosphere soil samples. Among them, 11 OTUs were specific for the rhizosphere soil habitat (Table 3). However, no OTUs associated with the root habitat were detected.

**Figure 3:**
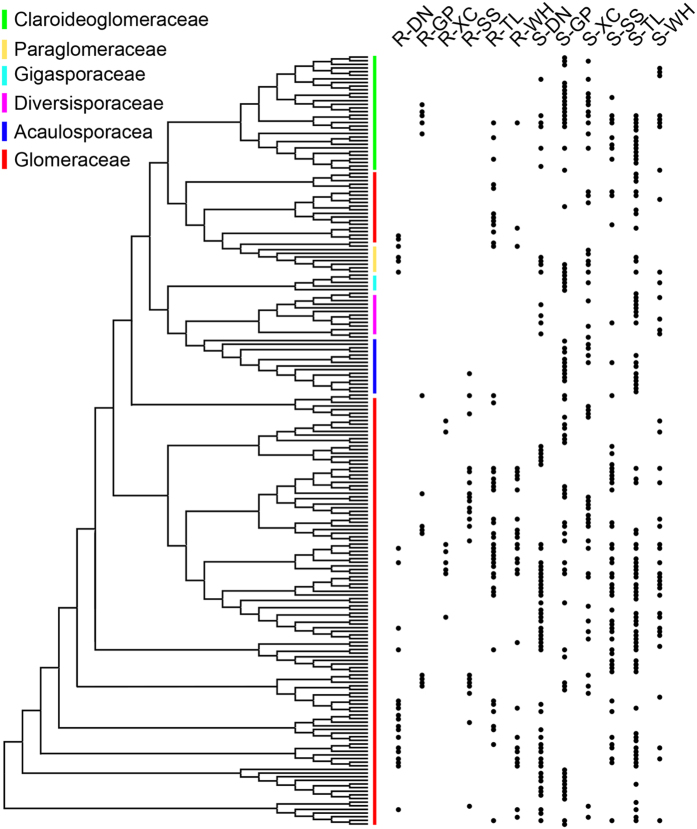
Phylogenetic tree of OTUs in different habitats. The tree contains all OTUs detected in this study. Dots at the right side of the tree indicate the presence of OTUs in the habitat types listed above the tips.

**Figure 4:**
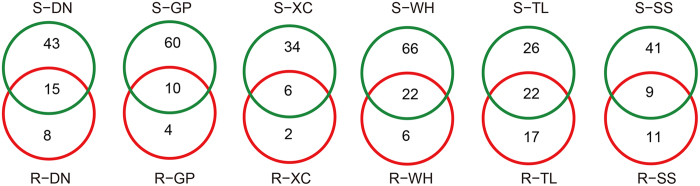
Venn diagram depicting OTUs that are shared or unique to root and soil samples.

In total, 210 OTUs were found in the AMF communities based on 97% species identity (Fig. 3). The phylogenetic placement of OTUs in different habitats was determined using the phylogenetic tree (Fig. 3). In general, the OTUs in rhizosphere soils were evenly scattered in the phylogenetic tree. However, most of AMF detected in root habitats were Glomeraceae while few AMF belonging to Acaulosporacea, Diversisporaceae or Gigasporaceae were found. Claroideoglomeraceae, Paraglomeraceae were identified in the roots of DN, GP and TL. Although the assemblages of AMF communities were markedly different, most of the OTUs in root samples were shared by the corresponding soil samples from the same site (Fig. 4).

### Relationship between AMF community structure and environmental factors

**Figure 5:**
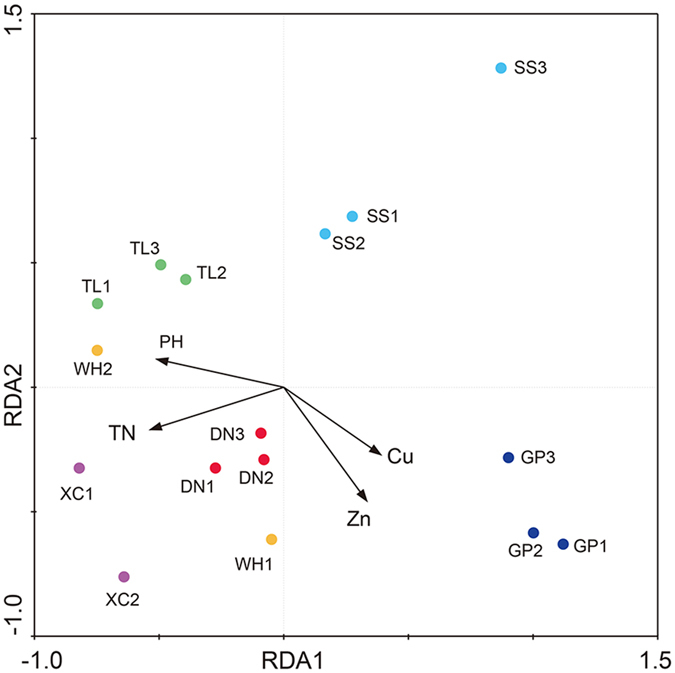
Redundancy analysis (RDA) of AMF communities and soil properties. The first two axes explained 31.7% of the total variance. Only soil variables with significant effects in Monte Carlo tests (P < 0.05) are shown.

Relationships of AMF richness (indicated by the ACE index) and phylogenetic diversity (revealed by the Faith’s index) with plant, soil and climate variables were analyzed (Supplementary Table S3). We found that the soil NH4+-N level correlated negatively with the AMF richness and phylogenetic diversity (Kendall test, P < 0.05). RDA, the multivariate analysis based on constrained ordination, was applied to analyze the influence of soil properties on the AMF community composition in *C. ambrosioides* rhizospheres (Fig. 5). The first two RDA components explained 31.7% of the total variation. The results showed that total N (P = 0.007), pH (P = 0.029), Zn (P = 0.033), and Cu (P = 0.047) explained the AMF community in soil habitats most and had a significant correlation between each variable and the ordination scores (Fig. 5).

**Figure 6:**
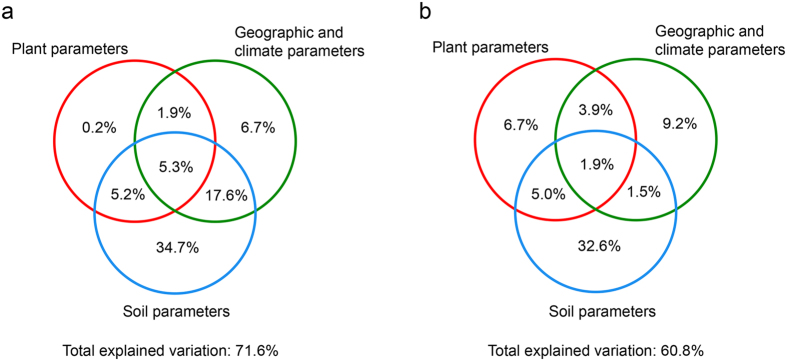
The contributions of environmental factors to the variation of AMF communities based on a stepwise distance-based redundancy analysis (db-RDA). (**a**) AMF community composition based on Bray-Curtis dissimilarity matrices. (**b**) AMF phylogenetic structure based on Unifrac distance matrices. The contributions of three groups of environment factors (soil, geographic and climate, and plant variables) or the combined groups were determined separately.

To further analyze the relative importance of soil characters, plant parameters, geographic distance, and climate properties in predicting AMF community composition and phylogenetic structure, a stepwise distance-based redundancy analysis (db-RDA) was carried out. In contrast to traditional RDA, an advantage of db-RDA is that it enables the inclusion of environmental factors and the testing of their interaction using non-Euclidean distance matrices. Totally, 71.6% and 60.8% of the variations of the soil AMF community composition and phylogenetic structure were explained by the whole set of the selected variables respectively (Fig. 6). A very large fraction of variations of AMF community composition (62.8%) and phylogenetic structure (41.0%) could be explained by soil properties, followed by geographic and climate variables (31.5% for community composition and 16.5% for phylogenetic structure respectively). The plant variables explained a relative small proportion of the variations in both community composition and phylogenetic structure.

Permutational multivariate analysis of variance (PerMANOVA) was also carried out to examine the relative importance of each single environmental factor to the AMF community composition and phylogenetic structure (Supplementary Table S4). pH showed weak associations with the AMF community composition and phylogenetic structure, while the soil available Cu content revealed significant associations with them. Besides, the PCNM vector (principal components of neighbor matrices, representing the spatial factors) and MAP (mean annual precipitation) showed significant associations to the AMF community composition. For the AMF community phylogenetic structure, soil total Cu, Zn, and Cd contents as well as MAT (mean annual temperature) were significantly correlated.

## Discussion

It is commonly accepted that more than 80% of terrestrial plants are colonized by AMF, with which they form associations^29^,^30^. The extraradical hyphal networks produced by AMF can alter plant physiology, enhance plant nutrient absorption and translocation, and increase plant resistance to HMs^31^,^32^,^33^. Root habitats showed a significantly smaller number of AMF OTUs compared to rhizosphere soil habitats, which was confirmed by the lower Chao1 and ACE richness indices of root samples. Similar results have previously been reported^18^,^34^. Besides, the PCoA based on the OTU composition showed that the samples from root and soil habitats at the same site clustered together, which was consistent with the hierarchical cluster analysis results, indicating similarities in the AMF community composition among root and soil samples from the same site and variation among samples from different sites. The indicator species analyses revealed 16 OTUs associated with rhizosphere soils while no OTUs with the root habitat were detected. Besides, most OTUs were shared by roots and rhizosphere soils from the same site, while few OTUs were shared among sites. These results indicated that AMF in rhizosphere soils could be considered to represent a pool of species, a fraction of which is recruited by plants^35^,^36^.

Xu *et al*. detected a strong influence of the plant community on AMF communities in soil, indicating preferences in plant-AMF associations^37^. These preferences have been observed on both local and global scale systems^36^,^38^,^39^, which might result from that host plants exhibited preferential allocation of photosynthates to more beneficial AMF partners^40^. Consistently, Saks *et al*. showed that root-colonizing AMF represent a phylogenetically clustered subset of AMF available in soil^41^. The conclusion is also proved in this study by the result that most of the AMF in root habitats were Glomeraceae. These results indicated that there might be niche preferences among AMF affecting AMF community composition^34^,^42^. Different to the study of AMF communities by Xu *et al*. which covered a wide range of vegetation types^37^, all soil samples were collected from rhizosphere of a single plant with similar size and age to reduce the plant factors affecting the AMF community in this study. Similar sampling strategy was also used for the study of AMF communities in semiarid Mediterranean soils^34^. We did not detect significant influences of the single plant on AMF communities in soils, indicating that the sampling strategy successfully reduce the biotic factors affecting the AMF distribution. Furthermore, by comparing influences of a single plant on AMF communities in roots and rhizosphere soils, we suggest that the effect of AMF communities in soils by plant communities might result from the different composition of plant communities in which each single plant shows the specific recruitment preference of AMF partners.

*Glomus* and *Rhizophagus* were the most abundant genera in root samples. Yang *et al*. reported that almost all of the sequences found in the roots of *Elsholtzia splendens* were *Glomus* species^43^. Long *et al*. revealed that although *Ambispora, Kuklospora*, and *Glomus* dominated in the rhizosphere soils of *Phytolacca americana*, only *Glomus* was detected in the roots^44^. Our results are in agreement with these previous studies. *Glomus* has been found to be dominant in various habitats, such as HM-polluted soils^45^, grassland soils^46^, and agricultural soils^47^. The *Rhizophagus* group is also a general AMF group that has been found in diverse host species and environments^48^,^49^,^50^. However, almost all of the fungi detected in R-DN and R-XC (root samples from DN and XC) were *Glomus*, the relative levels of which were much higher than those in soil habitats. On the contrary, the relative level of *Glomus* in R-GP (root samples from GP) was markedly lower than that in S-GP (soil samples from GP), while *Rhizophagus* was significantly more abundant in R-GP than S-GP. The *Rhizophagus* level was also higher in the root samples from TL and WH compared to the corresponding soil samples, while no obvious recruitment was detected for R-SS. These results indicated that the AMF recruitment preferences by *C. ambrosioides* differed among sampling sites. Several explanations for the differences in the AMF present in the roots and rhizosphere soils of the same plant have been proposed, including different AMF life strategies, differential sporulation dynamics, and seasonal changes in the AMF community^50^,^51^,^52^.

It has been shown that geographical distance influences AMF community at large spatial scales, especially in global-scale studies^37^. However, at local or landscape scales, soil abiotic factors are the key driver in shaping AMF community composition^10^,^11^. In our study, the geographic and climate parameters could independently explain a small fraction (<10%) of variances of AMF community composition and phylogenetic structure, while a large fraction of variances (>32%) could be explained independently by soil variables. These results revealed the complexity of factors regulating AMF communities, and the distribution patterns of AMF communities could not be completely explained by soil heterogeneity.

Despite high levels of Mn in soils of DN, GP and XC, no significant differences in the species diversity and richness of AMF communities were detected between these three samples and others. However, the richness indices of the R-GP and R-XC were markedly lower than those of the other samples, revealing that Mn affects the AMF community to some extent. These results are similar to Wei *et al*., which reported that root colonization and AMF diversity were negatively correlated with soil Mn concentration^17^. These results suggest also that other environmental factors may affect the AMF community and the effects of Mn on the AMF community may be confounded by those of other factors. Indeed, various factors influence the AMF community^41^. For example, Yang *et al*. showed that HM contamination is not the only soil parameter that affects the AMF community, and soil pH, Pb, Zn, Cd, and OM levels also have a great influence^18^; Wei *et al*. concluded that changes in AMF diversity and colonization are not solely attributed to soil Mn concentration, while soil properties, especially N concentration, were also closely related to it^53^.

In this study, pH was found to be a significant factor influencing AMF communities. Similarly, some studies concluded that pH is the key environmental factor shaping AMF communities^54^. Soil acidity is one of the most important drivers of microbial communities, particularly for AMF^11^,^55^. Soil pH can directly change the physiological status of indigenous AMF, alter their ecological niches, and it could also indirectly influences the AMF community by regulating soil nutrient bioavailability, and impacting the mobility and sorption of metals. Several studies have reported strong effects of soil Zn and Cu levels on AMF abundance and diversity in soils^18^,^34^,^56^. These nutrients play important roles in plant metabolic processes, and their uptake is influenced by AMF^57^,^58^. It has been shown that total N is related to changes in the composition of soil microbial communities^59^. Avio *et al*. showed N-fertilization was the main factor shaping AMF communities^60^ while Van Diepen *et al*. revealed N addition significantly altered the AMF community structure^61^. The lack of nitrogen could cause inhibition of sporulation^62^, while high N availability can change nutritional processes in AMF and alter the abundance of AMF phylotypes^63^. Besides, the N need of a plant could facilitate colonization by AMF. These features may explain the reasons why total N, pH, Zn, and Cu affect AMF community in *C. ambrosioides* rhizosphere.

## Conclusion

The AMF communities of the roots and rhizosphere soils of *C. ambrosioides* in six areas were investigated. Both habitats (root or rhizosphere soil) and soil properties are the key environmental factors affecting AMF communities with *C. ambrosioides*. Total N, pH, Zn, and Cu levels play significant roles in triggering AMF populations. Overall, *C. ambrosioides* rhizospheric AMF communities and their influencing factors were illustrated, which contributed a better understanding of the AMF community shaping and related ecological factors.

## Methods

### Root and soil sampling

Samples were collected at six sites in Yunnan, Anhui, and Guangxi Provinces, China. Two sites in Yunnan province were located in Dounan Town (DN, 23°36′ N, 103°41′ E), Yanshan County, and Xincheng Town (XC, 23°38′ N, 102°27′ E), Shiping County. Three sites were located in Anhui Province, including Tongling (TL, 30°49′ N, 117°51′ E), Wuhu (WH, 31°19′ N, 118°26′ E) and Susong (SS, 30°9′ N, 116°10′ E) Counties. Another site was located in Guiping County (GP, 23°31′ N, 110°16′ E), Guanxi province. A single plant, *C. ambrosioides*, was investigated to reduce the number of biotic factors affecting the AMF community. All samples were collected in August 2015. Three healthy, similarly sized plants were randomly selected and collected at each of the six sites. Root systems were carefully excavated. Using clean tweezers and a brush, the soils bound to the surface of roots were carefully removed. The removed soils were defined as rhizosphere soil samples for DNA extraction. Then the roots were wrapped in tissue paper and stored in sterile Ziploc bags containing silica gel. Topsoil samples (0–15 cm depth) were collected from beneath each plant in at least three directions for chemical property analysis. To remove aboveground plant materials, roots, and stones, the mixed and homogenized soil samples were passed through a 2 mm sieve. After packing in sterile Ziploc bags, these soil samples were transported to the laboratory and stored at 4 °C to analyze physicochemical properties or at −80 °C for DNA extraction.

### Soil physicochemical properties

Soils were air-dried at room temperature, ground into a powder, and passed through a 0.15 mm nylon sieve. The soil pH was determined using a glass electrode with soil suspended in 0.01 M CaCl_2_ (soil: solution ratio, 1:5). The nitrate N (NO_3_^−^-N), ammonia N (NH_4_^+^-N), and soil organic matter (OM) levels in the soil samples were determined according to our previous study^59^. Available K in soil was extracted with ammonium acetate^64^, and then determined by flame photometry (AAS novAA 400, Analytik Jena AG, Jena, Germany). Using sodium bicarbonate, the available P in soil was extracted and measured using the molybdenum blue method according to Watanabe and Olsen^65^. The total N level in soil was determined using the micro-Kjeldahl method^66^, and measured using a continuous flow analyzer (AA3, Bran + Luebbe, Hamburg, Germany). Soil samples for analyzing the levels of total P, total K, and HM (Mn, Zn, Cd, Cu, and Pb) were dried at 105 °C for 6 h, passed through a 0.15 mm nylon sieve, and then digested in a mixture of HNO_3_ and HClO_4_ (4:1, v/v)^67^. The total concentrations of K, P, Mn, Cu, and Zn were determined by inductively coupled plasma optical emission spectrometry (ICP-OES; 710series, Agilent Technologies, Palo Alto, CA, USA), and inductively coupled plasma mass spectrometry (ICP-MS; NexION 300X, Perkin Elmer, Norwalk, CT, USA) was used to measure the Cd and Pb concentrations. To analyze available HM, 20 mL modified Morgan’s solution (1 M ammonium acetate; pH 4.8) was added to 4 g soil in 100 mL Erlenmeyer flasks. The mixture was shaken on a rotary shaker at 150 rpm for 15 min, and then clear solutions were obtained by filtering through filter paper. The extracts were analyzed in terms of HM concentrations by ICP-MS^59^. The chemical properties of three soil sample replicates were analyzed independently.

### DNA extraction and PCR amplification

Soil DNA was extracted from 0.5 g soil using a FastDNA SPIN Kit following the manufacturer’s instructions (MP Biomedicals, Santa Ana, CA, USA). Root DNA was extracted from 0.1 mg roots using the Plant DNA Extraction Kit (Tiangen Biotech, China) according to the manufacturer’s instructions. In total, 36 samples comprising 18 soil samples and 18 root samples were subjected to DNA extraction. After dissolving in 50 μL TE buffer and quantifying by spectrophotometry, the extracted DNA was stored at −20 °C for further analysis. A conserved AMF-specific primer set, AMV4.5NF (5′-AAGCTCGTAGTTGAATTTCG-3′)/AMDGR (5′-CCCAACTATCCCTATTAATCAT-3′), was used to amplify the partial AMF 18S rRNA gene fragment^68^. A 6 bp error-correcting barcode was included in the reverse primer to characterize the samples^55^. The polymerase chain reaction (PCR) was carried out in a 20 μL mixture, including 10 ng DNA template, 0.4 μL FastPfu Polymerase (TransGen Biotech, Beijing, China), 0.8 μL each 5 μM primer, 2 μL 2.5 mM dNTPs, 4 μL 5× FastPfu Buffer, and 0.2 μL bovine serum albumin (BSA; Takara Biotechnology, Dalian, China). The PCR conditions were as follows: 95 °C for 3 min; 28 cycles of denaturation at 95 °C for 30 s, primer annealing at 55 °C for 30 s, and extension at 72 °C for 45 s, followed by a final extension for 10 min at 72 °C.

### Illumina MiSeq sequencing

To reduce potential early-round PCR errors, three independent PCR products for each sample were combined to construct a PCR amplicon library. The PCR products were subjected to agarose gel electrophoresis and purified using an AxyPrep DNA Gel Extraction Kit (Axygen Biosciences, Union City, CA, USA) according to the manufacturer’s protocol. Then the amplified DNA was quantified using QuantiFluor™-ST (Promega, Madison, WI, USA). The purified amplicons were paired-end (PE) sequenced on an Illumina MiSeq platform (Shanghai Biozeron Biotechnology Co., Ltd, Shanghai, China) according to standard protocols. In total, 36 sequencing libraries were constructed and independently sequenced.

### Processing of sequencing data

The raw Illumina sequencing data were quality-filtered using the Trimmomatic software^69^. The reads were truncated at any site that received an average quality score <20 over a 50 bp sliding window, and the truncated reads shorter than 50 bp were discarded. Then, PE reads were assembled according to their overlap sequence with a minimum overlap length of 10 bp, discarding reads that could not be assembled. Sequences that contained more than one ambiguous character or two nucleotide mismatches in the primers, and those with a mismatch ratio within the overlap region of more than 0.2 were removed. The clean sequences were analyzed using the QIIME pipeline^70^. Chimeric sequences were identified and removed using UCHIME^71^. Operational taxonomic unit (OTU) grouping was performed using UPARSE at 97% similarity^72^, and then the representative sequences obtained for each OTU were assigned to taxonomic data using the RDP classifier at a 70% threshold^73^. The rarefaction curves, indices of ACE and Chao1, and Shannon diversity were analyzed using the Mothur software^74^. The Bray–Curtis distances were calculated using the QIIME pipeline^70^, and a principal coordinate analysis (PCoA) was performed using R (http://www.r-project.org/) based on the Bray–Curtis similarities. A Venn diagram based on unique and shared OTUs was produced using R to characterize the differences and similarities among the AMF communities. The indicator species analyses were performed to test whether there were specific OTUs associated with the rhizosphere soil habitat or root habitat, and the indicator value index was used to measure the associations^75^. The analyses were performed using the indcspecies package implemented in R with a permutation test (999 permutations)^76^. To examine the relationships between AMF communities and soil properties, redundancy analysis (RDA) was applied. This analysis was conducted with CANOCO for Windows^77^ with 999 permutations of Monte Carlo permutation tests.

### Phylogenetic analysis

A neighbor-joining tree containing the type sequences of all OTUs was constructed using MEGA v6.0 with 1000 replicates^78^. All sequences were aligned, concatenated, and manually adjusted using Geneious Pro v4.8.3 (http://www.geneious.com/). The best-fit model for the datasets was selected using jModelTest v2^79^.

### Statistical analyses

PCNM vectors were calculated using the ‘pcnm’ function of the ‘vegan’ package with the R language. Prior to the calculation, GPS coordinates were converted to UTM coordinates in kilometers. PCNM vectors were used as explanatory spatial variables for db-RDA^80^. The weighted Unifrac distance matrix was used as a measure to determine the AMF phylogenetic structure, which was calculated using ‘unifracs’ function of ‘GUniFrac’ package with the R language. db-RDA^81^, which is known as constrained analysis of principal coordinates, was used to investigate the variations of AMF communities that were attributable to environmental factors. By a stepwise db-RDA, the contributions of environmental factors to the variation of soil AMF communities were summarized using Bray-Curtis dissimilarity and Unifrac distance matrices separately. The forward-selection for the environmental factors in three groups (soil, geographic and climate, and plant variables) was performed independently with the adjR2thresh stopping criterion^82^, and then the contribution of each of the groups or the combined groups was determined. The amount of variances explained by the individual and combined groups was tested using Monte Carlo permutation tests (999 permutations). The stepwise db-RDA was performed using ‘capscale’ function of ‘vegan’ package with the R language. PerMANOVA for the relationship between AMF community composition and each environmental variable was carried out separately based on Bray-Curtis dissimilarity distance and weighted Unifrac distance matrices using ‘adonis’ function of ‘vegan’ package with the R language. Tukey’s and Kendall test was used for multiple comparisons (*P* < 0.05), which were performed using SPSS version 19.0 (SPSS Inc., Chicago, IL, USA).

## Additional Information

**How to cite this article:** Xu, X. *et al*. The influence of environmental factors on communities of arbuscular mycorrhizal fungi associated with *Chenopodium* *ambrosioides* revealed by MiSeq sequencing investigation. *Sci. Rep.* **7**, 45134; doi: 10.1038/srep45134 (2017).

**Publisher's note:** Springer Nature remains neutral with regard to jurisdictional claims in published maps and institutional affiliations.

## Supplementary Material

Supplementary Information

## Figures and Tables

**Table 1:** Chemical properties of sampled soils.

	DN	GP	XC	SS	TL	WH
pH	7.62 ± 0.11(a)	5.62 ± 0.51(b)	7.37 ± 0.25(a)	7.04 ± 0.07(a)	7.40 ± 0.06(a)	7.52 ± 0.02(a)
OM (g/kg)	9.86 ± 2.66(b)	4.34 ± 1.11(b)	6.87 ± 2.80(b)	7.11 ± 0.27(b)	19.74 ± 2.20(a)	16.30 ± 1.53(a)
NH_4_^+^-N (mg/kg)	26.74 ± 1.92(a)	18.39 ± 13.65(ab)	24.64 ± 0.60(a)	21.11 ± 1.49(a)	4.66 ± 1.99(bc)	2.31 ± 0.18(c)
NO_3_^−^-N (mg/kg)	2.61 ± 0.52(a)	3.17 ± 0.80(a)	18.54 ± 15.69(a)	1.65 ± 0.15(a)	3.63 ± 2.35(a)	2.12 ± 0.99(a)
TN (g/kg)	0.08 ± 0.02(bc)	0.04 ± 0.01(bc)	0.17 ± 0.05(a)	0.02 ± 0.01(c)	0.12 ± 0.03(ab)	0.12 ± 0.04(ab)
TP (mg/kg)	1850.0 ± 242.7(a)	1820.6 ± 196.1(a)	1654.6 ± 1016.4(ab)	258.5 ± 156.2(c)	273.0 ± 153.2(c)	471.6 ± 246.2(bc)
TK (mg/kg)	4586.3 ± 1166.2(ab)	4856.3 ± 1156.9(ab)	12688.0 ± 7063.2(a)	2564.6 ± 511.1(b)	2514.6 ± 550.0(b)	1878.0 ± 2393.5(b)
TMn (mg/kg)	38142.0 ± 9533.6(a)	41772.6 ± 13937.3(a)	51005.3 ± 9309.9(a)	166.0 ± 71.1(b)	436.6 ± 72.2 (b)	336.6 ± 218.5(b)
TCu (mg/kg)	51.35 ± 7.74(ab)	111.34 ± 37.21(a)	105.49 ± 28.89(a)	15.38 ± 0.30(b)	14.55 ± 3.65(b)	23.22 ± 35.72(b)
TZn (mg/kg	121.67 ± 9.07(bc)	346.67 ± 22.85(a)	217.33 ± 53.12(b)	23.97 ± 4.53(c)	28.65 ± 18.71(c)	91.99 ± 76.38(c)
TCd (mg/kg)	1.01 ± 0.27(abc)	3.12 ± 1.29(a)	2.26 ± 0.62(ab)	0.34 ± 0.04(c)	0.23 ± 0.05(c)	0.37 ± 0.08(c)
TPb (mg/kg)	17.23 ± 1.52(a)	32.56 ± 45.55(a)	69.92 ± 53.19(a)	18.85 ± 3.50(a)	11.01 ± 2.03(a)	20.99 ± 18.60(a)
AP (mg/kg)	26.49 ± 7.25(a)	44.38 ± 22.61(a)	37.75 ± 3.71(a)	29.25 ± 3.12(a)	44.19 ± 6.23(a)	42.78 ± 14.43(a)
AK (mg/kg)	422.33 ± 265.36(a)	142.33 ± 22.23(a)	220.67 ± 11.02(a)	426.33 ± 121.13(a)	254.67 ± 49.69(a)	533.67 ± 278.69(a)
ACu (mg/kg)	4.28 ± 2.17(a)	1.27 ± 0.23(b)	1.26 ± 0.71(b)	2.75 ± 0.13(ab)	4.88 ± 0.13(a)	4.46 ± 0.46(a)
AZn (mg/kg)	7.54 ± 2.79(a)	5.53 ± 1.17(a)	6.62 ± 2.92(a)	8.07 ± 1.19(a)	9.82 ± 1.21(a)	27.33 ± 21.01(a)
ACd (mg/kg)	0.43 ± 0.01(ab)	0.43 ± 0.03(ab)	0.24 ± 0.03(c)	0.53 ± 0.04(ab)	0.53 ± 0.01(a)	0.42 ± 0.08(b)
AMn (mg/kg)	937.07 ± 797.36(a)	275.86 ± 95.28(a)	64.71 ± 66.75(ab)	6.62 ± 0.96(b)	50.90 ± 4.73(ab)	30.27 ± 6.67(b)

Values are means followed by standard error. Different letters indicated statistically significant differences (*P* < 0.05) according to the Tukey test. T, total; A, available.

**Table 2:** Richness estimators and diversity indices at a 97% identity threshold.

Sample	Reads	OTU	ACE	Chao1	Shannon	Coverage
R-DN	29599 ± 1487	13.7 ± 7.4	15.7 ± 8.3 (b)	14.7 ± 8.3 (b)	0.92 ± 0.68 (a)	0.9999
R-GP	29134 ± 8882	5.7 ± 1.5	3.3 ± 3.1 (b)	5.7 ± 1.5 (b)	0.44 ± 0.42 (a)	1.0000
R-XC	8582 ± 542	4.0 ± 2.8	3.5 ± 3.4 (b)	4.0 ± 2.8 (b)	0.36 ± 0.35 (a)	0.9999
R-SS	13818 ± 1228	11.0 ± 4.0	11.7 ± 4.2 (b)	11.0 ± 4.0 (b)	0.75 ± 0.46 (a)	1.0000
R-TL	35005 ± 4388	19.7 ± 15.0	22.3 ± 16.2 (a)	21.7 ± 16.6 (a)	0.89 ± 0.85 (a)	0.9999
R-WH	22251 ± 4921	16.0 ± 12.7	17.5 ± 13.4 (b)	17.0 ± 14.1 (b)	1.56 ± 0.40 (a)	0.9999
S-DN	29173 ± 8693	30.7 ± 18.3	28.3 ± 24.8 (a)	32.3 ± 17.2 (a)	1.53 ± 0.54 (a)	0.9999
S-GP	19072 ± 3919	35.0 ± 18.0	35.3 ± 18.2 (a)	35.0 ± 18.0 (a)	2.08 ± 0.80 (a)	0.9999
S-XC	4822 ± 3865	20.5 ± 3.5	21.0 ± 4.2 (a)	21.0 ± 4.2 (a)	1.61 ± 0.04 (a)	0.9995
S-SS	19193 ± 5791	24.3 ± 8.4	26.0 ± 7.2 (a)	24.3 ± 8.4 (a)	1.54 ± 0.08 (a)	0.9999
S-TL	23156 ± 8602	29.0 ± 5.6	34.3 ± 9.3 (a)	35.3 ± 10.3 (a)	1.70 ± 0.52 (a)	0.9998
S-WH	31323 ± 4580	55.5 ± 16.3	61.0 ± 21.2 (a)	60.0 ± 19.8 (a)	2.05 ± 0.81 (a)	0.9998

Values are means followed by standard error. Different letters indicated statistically significant differences (*P* < 0.05) according to the Tukey test. R, root samples; S, soil samples.

**Table 3:** OTUs associated with the rhizosphere soil habitat.

OUT	Taxon	Probability		Indicator value index	P
		A	B		
1	*Funneliformis mosseae*	0.997	0.625	0.789	0.002
2	*Glomus sp*. WUM3	0.996	0.563	0.748	0.004
3*	*Claroideoglomus luteum*	0.853	0.625	0.730	0.018
4*	*Septoglomus viscosum*	1.000	0.500	0.707	0.005
5	*Funneliformis mosseae*	0.993	0.500	0.705	0.003
6*	*Septoglomus viscosum*	1.000	0.438	0.661	0.01
7*	*Claroideoglomus claroideum*	1.000	0.375	0.612	0.017
8*	*Paraglomus occultum*	1.000	0.375	0.612	0.018
9*	*Funneliformis mosseae*	1.000	0.375	0.612	0.01
10*	*Glomus sp*. clA	1.000	0.375	0.612	0.015
11*	*Glomus sp*. ZJ	1.000	0.313	0.559	0.045
12*	*Septoglomus viscosum*	1.000	0.313	0.559	0.035
13	*Claroideoglomus claroideum*	1.000	0.313	0.559	0.043
14*	*Glomus sp*. CaAIM7	1.000	0.313	0.559	0.048
15*	*Septoglomus viscosum*	1.000	0.313	0.559	0.049
16	*Septoglomus viscosum*	1.000	0.313	0.559	0.035

A is the probability that the surveyed site belongs to a given environment, given the fact that the species has been found; B is the probability of finding the species in sites belonging to a given environment. Asterisks refer to OTUs specific for rhizosphere soil habitats.
