# Supplementary material for: The influence of environmental factors on communities of arbuscular mycorrhizal fungi associated with Chenopodium ambrosioides revealed by MiSeq sequencing investigation
Source: Sci Rep. 2017 Mar 22;7:45134. doi: 10.1038/srep45134 (PMC5361092; doi:10.1038/srep45134)
Supplement: Supplementary Information [file srep45134-s1.pdf]

Supplementary information

The influence of environmental factors on communities of arbuscular mycorrhizal fungi associated with *Chenopodium ambrosioides* revealed by MiSeq sequencing investigation

Xihui Xu[1], Chen Chen[1], Zhou Zhang[1], Zehua Sun[1], Yahua Chen[1], Jiandong Jiang[1], Zhenguo Shen[1]*

[1] *College of Life Sciences, Nanjing Agricultural University, Nanjing 210095, People's Republic of China*

* Corresponding author

Email: zgshen@njau.edu.cn

Tel: 0086- 025-84396391

Fax: 0086-025-84396391

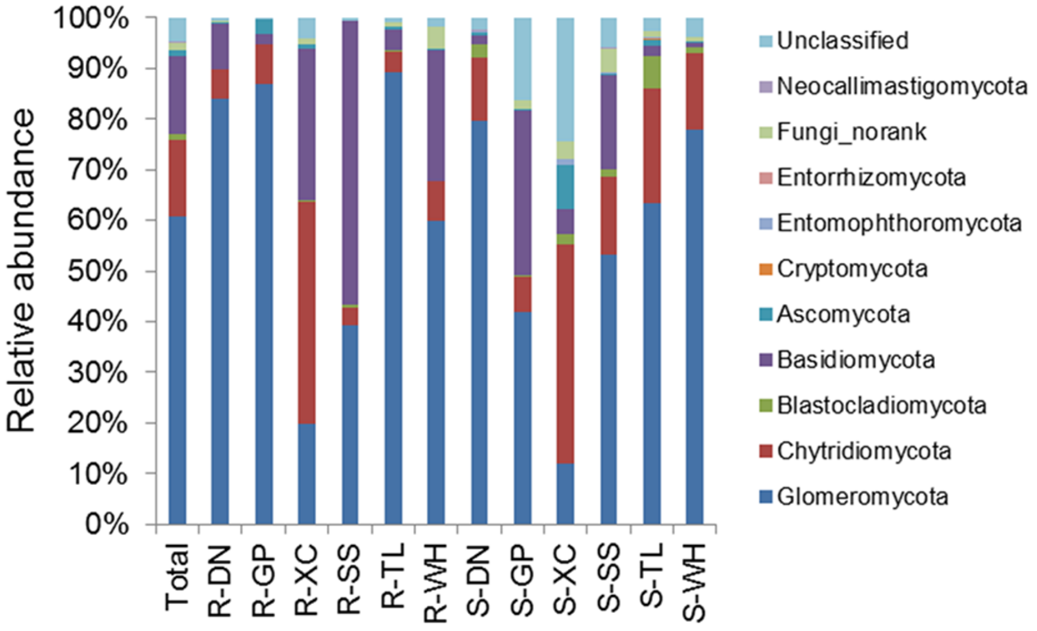

Supplementary Figure S1. Proportional distributions of sequences in each fungal phylum detected using the primer set AMV4.5NF/AMDGR in all samples.

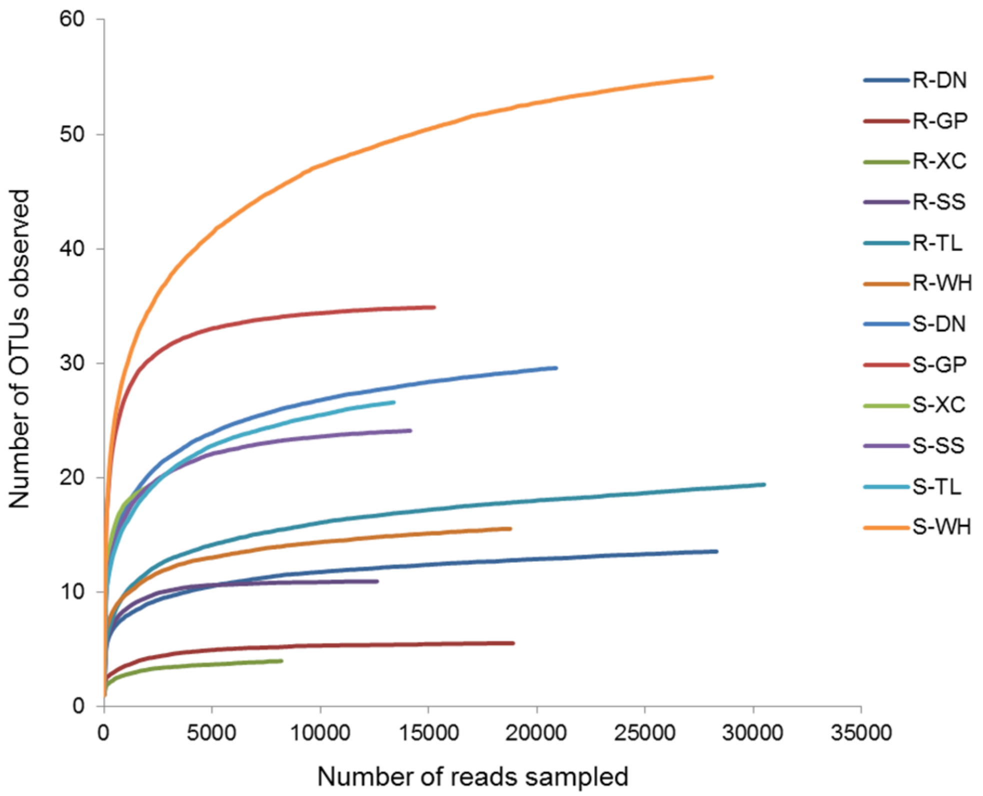

Supplementary Figure S2. Rarefaction curves of OTU numbers at a 97% similarity level. R, root samples; S, soil samples.

Table S1 Location, soil physical properties, plant parameters and climate characters of sampling sites. EC, electric conductivity; MAP, mean annual precipitation; MAT, mean annual temperature.

	TL	WH	SS	XC	DN	GP
Longitude	117.8544	118.4446	116.1676	102.4578	103.6937	110.2791
Latitude	30.83022	31.31743	30.15926	23.80824	23.59842	23.52772
EC	197.80±25.49	223.80±65.79	126.20±17.05	1315.00±667.91	181.60±39.71	98.82±27.67
Sand(%)	55.84±6.38	79.70±5.35	67.01±7.58	87.78±6.49	77.88±4.72	85.04±2.33
Silt(%)	40.78±5.44	18.07±5.30	28.36±5.67	9.09±6.34	19.18±2.26	12.07±1.66
Clay(%)	3.38±1.52	2.23±1.28	4.63±2.86	3.13±1.69	2.94±2.51	2.88±0.94
Moisture(%)	5.73±3.68	12.78±2.42	9.88±0.77	10.69±1.75	6.83±3.03	10.17±3.84
Plant age (annual herb)	Flowering period	Flowering period	Flowering period	Flowering period	Flowering period	Flowering period
Plant height(cm)	34±5	37±4	37±5	43±7	45±9	78±11
Plant biomass(g)	1.51±0.33	0.97±0.24	1.38±0.37	1.92±0.41	1.28±0.19	2.75±0.56
Elevation(m)	96	9	79	1292	1711	115
MAT(℃)	16.5	16.3	17	19.5	17.8	21.9
MAP(mm)	1256	1173	1403	1041	954	1601

Table S2 Statistics of the effective tag sequences of samples.

Sample	Sequences	Bases(bp)	Average Length(bp)
R-DN1	30019	6463991	215.33
R-DN2	32967	7100313	215.38
R-DN3	43339	9325848	215.18
R-GP1	35461	7666092	216.18
R-GP2	30778	6646262	215.94
R-GP3	34685	7545168	217.53
R-XC1	43831	9471330	216.09
R-XC2	42787	9442829	220.69
R-XC3	38244	8678351	226.92
R-SS1	40461	8729866	215.76
R-SS2	33138	7160268	216.07
R-SS3	32523	7007691	215.47
R-TL1	39093	8475905	216.81
R-TL2	40592	8765578	215.94
R-TL3	38734	8390574	216.62
R-WH1	42109	9166852	217.69
R-WH2	32555	7033307	216.04
R-WH3	35882	7855678	218.93
S-DN1	33380	7226078	216.48
S-DN2	32489	7080583	217.94
S-DN3	44598	9624671	215.81
S-GP1	35823	7945800	221.81
S-GP2	42982	9609398	223.57
S-GP3	33263	7319434	220.05
S-SS1	34142	7484047	219.2
S-SS2	41701	9149457	219.41
S-SS3	33009	7296450	221.04
S-TL1	37471	8123507	216.79
S-TL2	41389	8877831	214.5
S-TL3	31191	6811193	218.37
S-WH1	43558	9509095	218.31
S-WH2	37828	8171502	216.02
S-WH43	39975	8786641	219.8
S-XC1	43594	10080822	231.24
S-XC2	38491	8353997	217.04
S-XC3	35991	7968528	221.4

Table S3 Correlation of soil AMF community richness (based on ACE index) and phylogenetic diversity (based on Faith's index) with abiotic environmental variables and plant parameters revealed by Kendall tests. T, total; A, available; EC, electric conductivity; MAP, mean annual precipitation; MAT, mean annual temperature.

	ACE index		Faith's index	
	R2	P	R2	P
pH	-0.018	0.927	-0.050	0.787
OM(mg/kg)	0.201	0.292	0.100	0.589
NH_4^+-N(mg/kg)	-0.638	0.001**	-0.400	0.031*
NO_3^--N(mg/kg)	-0.149	0.436	-0.133	0.471
TN(mg/kg)	0.096	0.614	-0.133	0.471
AP(mg/kg)	0.131	0.492	-0.133	0.471
AK(mg/kg)	0.070	0.714	0.209	0.260
ACu(mg/kg)	0.246	0.199	0.226	0.224
AZn(mg/kg)	0.271	0.155	0.350	0.059
ACd(mg/kg)	0.185	0.335	0.151	0.417
AMn(mg/kg)	0.026	0.891	0.000	1.000
TK(mg/kg)	-0.306	0.109	-0.183	0.322
TMn(mg/kg)	-0.096	0.614	-0.133	0.471
TCu(mg/kg)	-0.166	0.384	-0.083	0.653
TZn(mg/kg)	0.026	0.891	0.000	1.000
TCd(mg/kg)	-0.158	0.409	-0.042	0.822
TPb(mg/kg)	-0.061	0.748	0.000	1.000
Plant biomass(g)	-0.088	0.646	-0.259	0.162
Plant height(cm)	-0.186	0.334	-0.059	0.752
MAT(℃)	-0.358	0.060	-0.133	0.471
MAP(mm)	0.088	0.646	0.126	0.499
EC	0.079	0.680	-0.033	0.857
Moisture(%)	0.061	0.748	0.150	0.418
Sand(%)	0.009	0.963	0.083	0.653
Silt(%)	0.114	0.551	-0.067	0.719
Clay(%)	-0.184	0.336	-0.133	0.471

Table S4. PerMANOVA analysis for the Correlation between soil AMF community composition (Bray-Curtis dissimilarity distance and Weighted Unifrac distance) and each environmental variables. T, total; A, available; EC, electric conductivity; MAP, mean annual precipitation; MAT, mean annual temperature; PCNM, principal coordinates of neighbor matrices, which is the explanatory variables of geographic distance predictors.

	Bray-Curtis		Unifrac	
	R2	P	R2	P
pH	0.140	0.094	0.110	0.076
NH4(mg/kg)	0.075	0.297	0.074	0.363
NO3(mg/kg)	0.037	0.661	0.076	0.290
OM(g/kg)	0.089	0.194	0.105	0.105
TN(mg/kg)	0.049	0.655	0.042	0.824
ACd(mg/kg)	0.043	0.699	0.077	0.304
ACu(mg/kg)	0.164	0.010*	0.140	0.017*
AK(mg/kg)	0.098	0.161	0.106	0.080
AMn(mg/kg)	0.095	0.065	0.079	0.236
AP(mg/kg)	0.063	0.430	0.074	0.321
AZn(mg/kg)	0.044	0.477	0.048	0.806
TCd(mg/kg)	0.096	0.178	0.140	0.023*
TCu(mg/kg)	0.094	0.184	0.138	0.015*
TZn(mg/kg)	0.098	0.156	0.138	0.029*
TK(mg/kg)	0.042	0.694	0.103	0.106
TMn(mg/kg)	0.115	0.067	0.094	0.166
TP(mg/kg)	0.157	0.008**	0.053	0.665
TPb(mg/kg)	0.038	0.699	0.085	0.215
Plant Biomass(g)	0.123	0.071	0.106	0.073
Plant Height(cm)	0.113	0.111	0.116	0.055
MAP(mm)	0.190	0.002**	0.079	0.275
MAT(℃)	0.116	0.075	0.158	0.006**
EC	0.047	0.573	0.064	0.452
PCNM1	0.132	0.015*	0.078	0.286
PCNM2	0.108	0.088	0.068	0.394
Moisture(%)	0.073	0.360	0.118	0.054
Sand(%)	0.080	0.317	0.099	0.130
Silt(%)	0.074	0.329	0.111	0.061
Clay(%)	0.058	0.542	0.040	0.845
